# A 2D-DIGE-based proteomic analysis reveals differences in the platelet releasate composition when comparing thrombin and collagen stimulations

**DOI:** 10.1038/srep08198

**Published:** 2015-02-03

**Authors:** Paula Vélez, Irene Izquierdo, Isaac Rosa, Ángel García

**Affiliations:** 1 Center for Research in Molecular Medicine and Chronic Diseases (CIMUS), Universidade de Santiago de Compostela, Santiago de Compostela, Spain; 2 Instituto de Investigación Sanitaria (IDIS), Santiago de Compostela, Spain; 3 Departament of Pharmacology, Faculty of Pharmacy, Universidade de Santiago de Compostela, Santiago de Compostela, Spain

## Abstract

Upon stimulation, platelets release a high number of proteins (the releasate). There are clear indications that these proteins are involved in the pathogenesis of several diseases, such as atherosclerosis. In the present study we compared the platelet releasate following platelet activation with two major endogenous agonists: thrombin and collagen. Proteome analysis was based on 2D-DIGE and LC-MS/MS. Firstly, we showed the primary role of thrombin and collagen receptors in platelet secretion by these agonists; moreover, we demonstrated that GPVI is the primary responsible for collagen-induced platelet activation/aggregation. Proteomic analysis allowed the detection of 122 protein spots differentially regulated between both conditions. After excluding fibrinogen spots, down-regulated in the releasate of thrombin-activated platelets, 84 differences remained. From those, we successfully identified 42, corresponding to 37 open-reading frames. Many of the differences identified correspond to post-translational modifications, primarily, proteolysis induced by thrombin. Among others, we show vitamin K-dependent protein S, an anticoagulant plasma protein, is up-regulated in thrombin samples. Our results could have pathological implications given that platelets might be playing a differential role in various diseases and biological processes through the secretion of different subsets of granule proteins and microvesicles following a predominant activation of certain receptors.

Platelets are small anucleate cells that play a fundamental role in haemostasis. Undesired platelet activation and formation of arterial thrombi are implicated in many diseases, such as myocardial infarction and stroke^1^. More recently, platelets have been also shown to play a role in other diseases and biological processes, such as angiogenesis, cancer metastasis, or immune response^2^. Once activated, platelets release a high number of proteins and other biomolecules, which is known as the releasate. During the last decade, a few groups have applied various proteomic approaches to study in detail the platelet releasate^3^,^4^,^5^,^6^. Platelets were primarily stimulated with thrombin; in some cases microvesicles were removed prior to analysis^3^ whereas in others not^5^,^6^. Besides providing a repertoire of platelet secreted proteins, the study of the platelet releasate has led to the identification of proteins relevant to disease. For example, Coppinger and colleagues found some platelet-released proteins in human atherosclerotic plaques, which indicates they could be contributing to the pathogenesis of atherosclerosis^3^. Moreover, the impact of aspirin in the platelet releasate was also studied by the same group, leading to the conclusion that aspirin has a general moderating effect on the amount of protein released regardless of the agonist^4^.

A recent report by Jonnalagadda and colleagues showed that platelet secretion is kinetically heterogeneous in an agonist-responsive manner^7^. In line with this, we tried to confirm the platelet secretome varies with the stimulus by comparing the platelet releasate following platelet activation with two major endogenous agonists: thrombin and collagen.

## Results

### The platelet releasate varies when comparing thrombin and collagen stimulations

**Figure 1:**
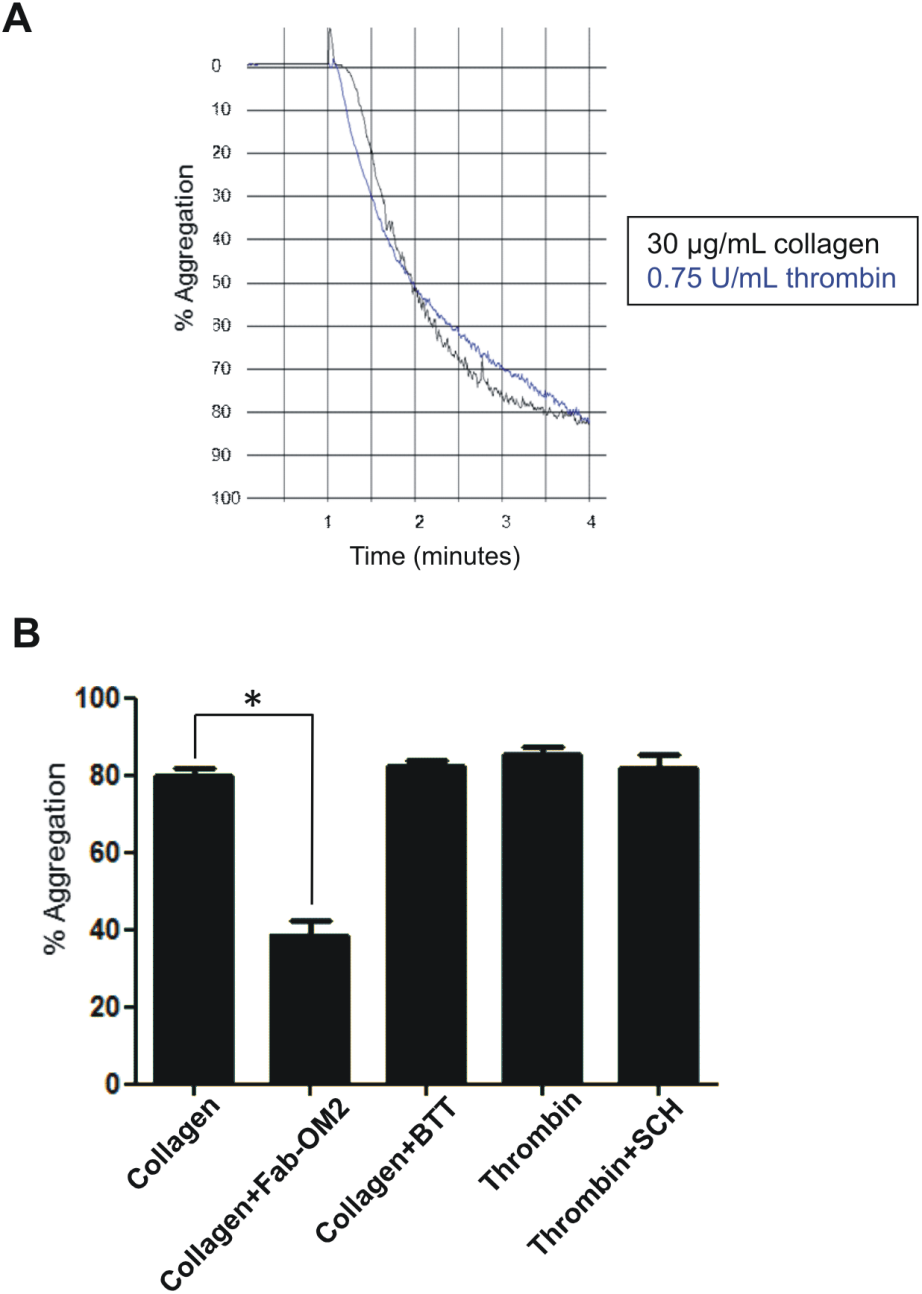
Effect of PAR-1, GPVI and α2β1 inhibitors on thrombin- and collagen-induced platelet aggregation. (A) Representative platelet aggregation profiles following platelet activation with 0.75 U/mL Thrombin (shown in blue) or 30 μg/mL collagen (shown in black). (B) Effect of PAR-1 inhibition on thrombin-induced platelet aggregation, and of GPVI and α2β1 inhibition on collagen-induced platelet aggregation. Washed human platelets were pre-incubated with the inhibitors for 5 min, then 0.75 U/mL thrombin or 30 μg/mL collagen were added to trigger platelet aggregation. Results are presented as mean ± SE (n = 4–6). *p<0.05 (Mann-Whitney test). Coll: collagen; Thr: thrombin; Fab-OM2: Fab fragment of the anti-GPVI monoclonal antibody OM2; BTT: BTT 3033; SCH: SCH 79797.

Platelets were isolated following a standardized procedure that minimizes contamination with other blood cells or plasma proteins, as well as activation during isolation^8^. Firstly, platelets were stimulated with the agonists at different concentrations to determine the minimum concentration needed to achieve maximum aggregation after 3 minutes. Aggregation of approximately 80% was achieved with the following concentrations: 0.75 U/mL of thrombin, and 30 µg/mL of collagen (Fig. 1A). Aggregation profiles were followed to make sure equal platelet aggregation levels were obtained with thrombin and collagen for each donor.

Besides the proteomic analysis, we decided to study the contribution of each receptor to platelet activation/aggregation by the above agonists at the final concentrations that were used. Interestingly, a report by Wu and colleagues showed a few years ago that thrombin-induced platelet activation, at doses above 0.5 U/mL, cannot be effectively inhibited by just blocking either single thrombin receptor pathway but by blocking them all (PAR-1, PAR-4, and GPIb)^9^. As a control, we tested the inhibition of the primary human thrombin receptor, PAR-1, and showed thrombin-induced platelet aggregation is not inhibited by the PAR-1 specific antagonist SCH 79797 (2 μM) (Fig. 1B). On the other hand, platelet activation with 10 μM TRAP-6 (SFLLRN) - specific PAR-1 agonist - was completely inhibited by 140 nM SCH 79797 (not shown).

Regarding collagen platelet activation, we inhibited the GPVI receptor by using the Fab fragment of the anti-GPVI monoclonal antibody, OM2, which works as specific antagonist of the receptor^10^. As expected, OM2 Fab fragment at a final concentration of 1 μg/mL was able of completely inhibiting platelet aggregation induced by 5 μg/mL of the GPVI specific agonist collagen-related peptide (CRP) (not shown). Regarding collagen-induced platelet aggregation, a maximum OM2 (Fab fragment) concentration of 51 μg/mL was able to inhibit aggregation by 30 μg/mL collagen by almost half (Fig. 1B). When testing a lower dose of collagen (3 μg/mL) we found that OM2 (Fab fragment) at 51 μg/mL was able to inhibit platelet aggregation by more than a 90% without affecting thrombin-induced aggregation (not shown). On the other hand, when the other main platelet collagen receptor, α2β1, was inhibited by using up to 13 μM BTT 3033 - a selective inhibitor of the integrin – no inhibition of collagen-induced platelet aggregation was observed at either 30 μg/mL or 3 μg/mL collagen dose (Fig. 1B, and not shown). These results demonstrate GPVI is the primary receptor responsible for collagen-induced platelet activation/aggregation.

After the aggregation studies, we proceeded with the isolation of the platelet releasate following 0.75 U/mL of thrombin, and 30 µg/mL of collagen activations. An initial screening by 1D-SDS-PAGE was done and obvious differences were observed when comparing thrombin and collagen-induced releasates (Supplementary Figure 1). Interestingly, we also obtained the platelet releasate under non-aggregating conditions - in the presence of integrilin (αIIbβ3 inhibitor) – and in the presence of inhibitors of secondary meditators, and analyzed it by 1D-SDS-PAGE just to check proteome profiles (Supplementary Figure 1). An evaluation of the stained 1D gel indicates differences between collagen and thrombin-induced releasates are bigger than when comparing the releasate for each of the agonists in the presence and absence of the inhibitors. Moreover, the presence of inhibitors did not have a significant impact on the amount of protein secreted following collagen or thrombin stimulations. Overall, our results suggest platelet secretion is mainly due to primary activation of collagen and thrombin receptors. We therefore decided to proceed with the proteomic analysis comparing the platelet secretomes following 0.75 U/mL thrombin, and 30 µg/mL collagen activations (without any inhibitor to better reproduce physiological conditions). The proteomic analysis was based on two-dimensional differential in-gel electrophoresis (2D-DIGE) and mass spectrometry, as indicated in the Methods section. We decided to analyze the whole releasate, including microvesicles, in order to maximize the information obtained. We focused on the pI 4–7 range of the proteome because it contains most platelet protein features. We used large gels (24 × 22 cm) to increase resolution of the analysis.

**Figure 2:**
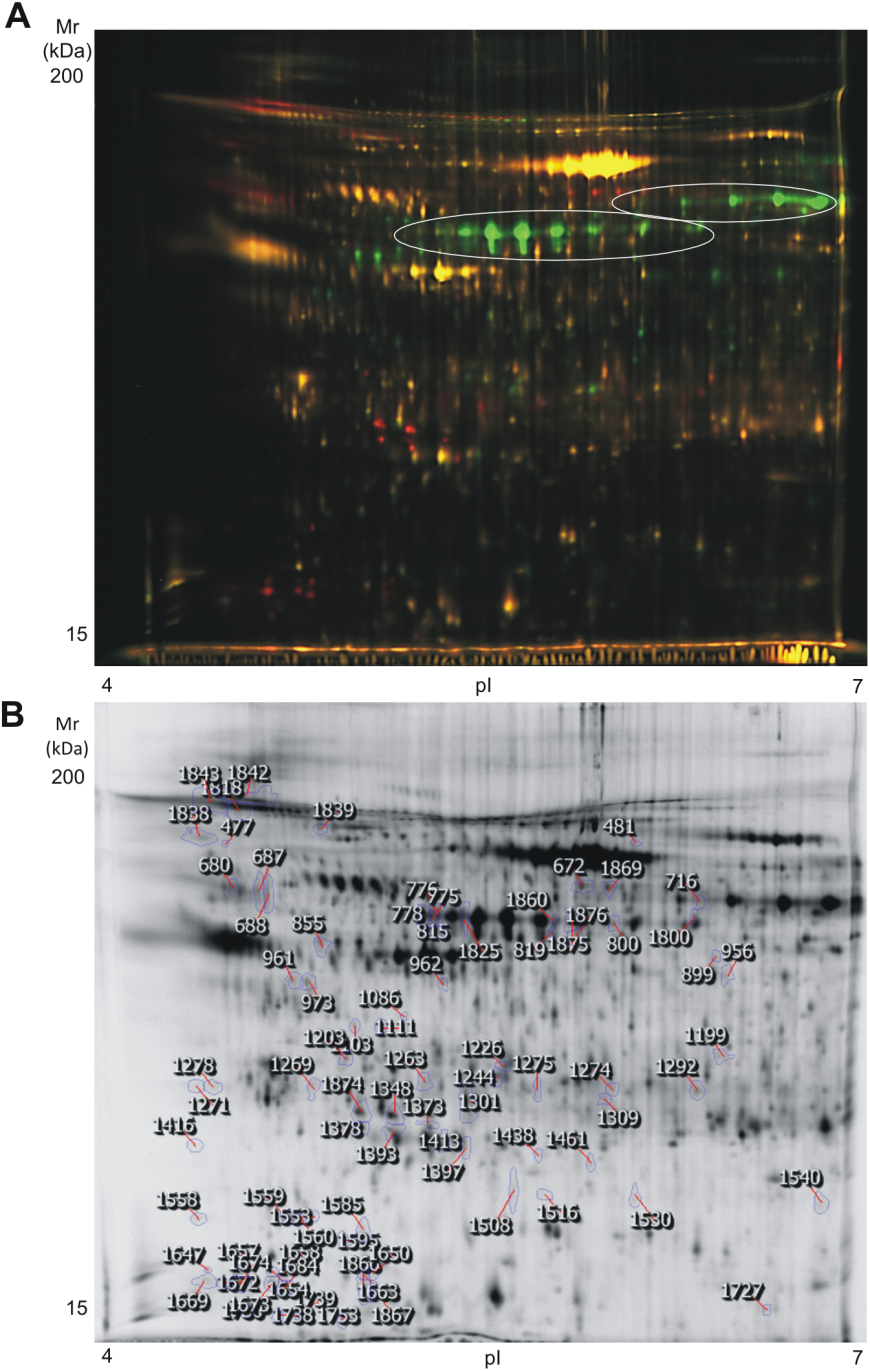
High-resolution 2D-DIGE proteome analysis of platelet releasate from collagen and thrombin stimulated platelets. Proteins were labeled with the corresponding Cy-dyes (see Methods section) and separated using isoelectric focusing (pH range 4–7, 24 cm) and 11% SDS-PAGE gels. (A) Representative image of the 2D-DIGE analysis where the fluorescence emission from Cy3 and Cy5 dyes is superimposed. Red-orange color spots are augmented in the releasate of thrombin-activated platelets whereas green color spots are augmented in the releasate of collagen-activated platelets. Main fibrinogen arrays are highlighted. (B) Representative image of the analysis in a gray scale with the differentially regulated spots (excluding fibrinogen) highlighted. Further information about protein identifications can be found in Table 1 and Supplementary Table 1.

In total, 1742 spots were detected per gel, from which 122 varied between the collagen-induced and the thrombin-induced releasate (fold change ≥2, p<0.05). Fifty-eight protein spots were up-regulated in the collagen group and 64 down-regulated. From those 122, 80 were successfully identified by mass spectrometry. It is necessary to bear in mind that 38 of the spots down-regulated in the thrombin-induced releasate corresponded to fibrinogen isoforms. Although other studies based on thrombin stimulation reported the presence of fibrinogen, in our case fibrinogen was almost absent. This is probably due to the higher concentration of agonist we used, which could cause the transformation of soluble fibrinogen into insoluble fibrin. Therefore, we decided to exclude spots containing exclusively fibrinogen from the analysis. After that, 84 differences remained (Fig. 2), 58 of which were increased in thrombin samples. Interestingly, 83 of the 84 differentially regulated protein spots had a p value lower than 0.01, which indicates a great level of significance. From those 84 differences, we successfully identified 42 protein features corresponding to 37 open-reading frames (Table 1; Supplementary Table 1). Remarkably, we did an analysis in parallel – using the same 2D-DIGE-based proteomic approach - comparing the collagen-induced platelet releasate in the presence and absence of 2 U/mL apyrase, 10 µM indomethacin (secondary mediators inhibitors), and 9 µM integrilin (αIIbβ3 inhibitor), and in line with the data mentioned above, we only found 18 differences (fold change ≥ 2, p < 0.05), a number much lower than when comparing collagen and thrombin releasates.

We checked the proteins identified searching for signal peptides and used the software Secretome P 2.0^11^ to confirm which proteins were released by a non-classical pathway or were referred to have a signal peptide. Forty-three per cent of the unique proteins identified had a signal peptide (i.e. secreted by a classical pathway); in fact, 94% of them were previously identified in the platelet releasate^3^,^4^,^5^,^6^,^12^,^13^. The remaining 57% were not secreted by the classical secretory pathways and were primarily part of secreted microvesicles.

**Figure 3:**
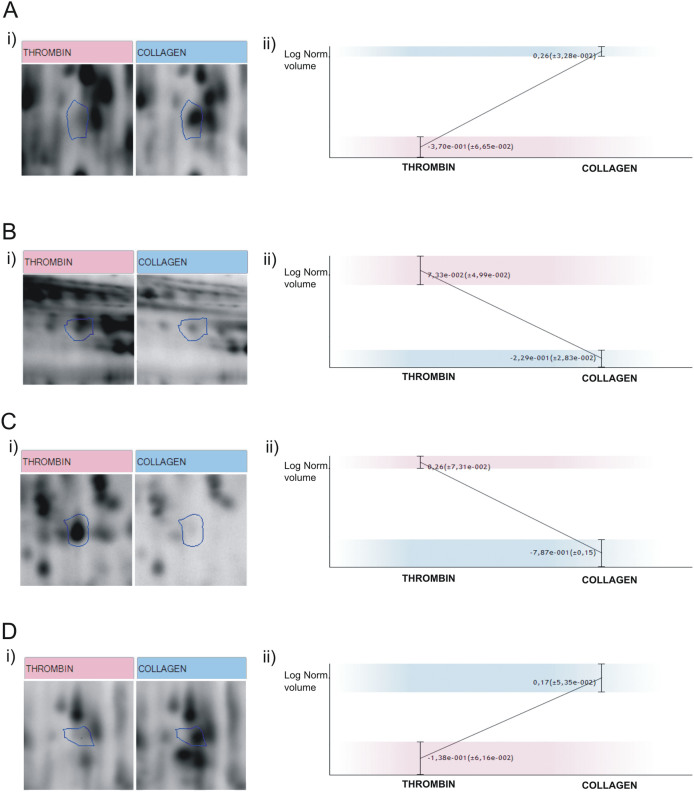
Selection of protein features differentially regulated between collagen- and thrombin-induced releasate. Enlargement of representative spots (i) with image analysis statistics (ii) for the following proteins: (A) MMRN1 (spot No. 1244); (B) PROS (spot No. 1839); (C) Coagulation Factor V(spot No. 1559); Thrombospondin-1 (spot No. 1226).

Some relevant proteins presenting differentially regulated features in the analysis include adhesive proteins (e.g. von Willebrand factor and thrombospondin-1) and clotting factors and their inhibitors (e.g. coagulation factor V, multimerin-1, and vitamin K-dependent protein S (PROS). A selection of differentially regulated protein features is shown in Figure 3. It is important to highlight many of the differences might be due to post-translational modifications (PTMs), primarily proteolysis. The latter is typically observed in the thrombin-induced releasate, as expected. An example of this is multimerin (MMRN1), a carrier protein for factor V/Va in platelets that is present in platelet α-granules but is undetectable in normal plasma^14^. We found that protein present in two spots up-regulated in the collagen-induced releasate (Fig. 3A). When doing a 1D western-blot analysis, we found MMRN1 present in several bands in a molecular weight range of 25 to 70 kDa (not shown). Although some of the bands vary between groups, overall there are no significant differences in total MMRN1 between collagen and thrombin-induced releasates, which suggests differences are simply due to PTMs.

### PROS is up-regulated in the thrombin-induced releasate

**Figure 4:**
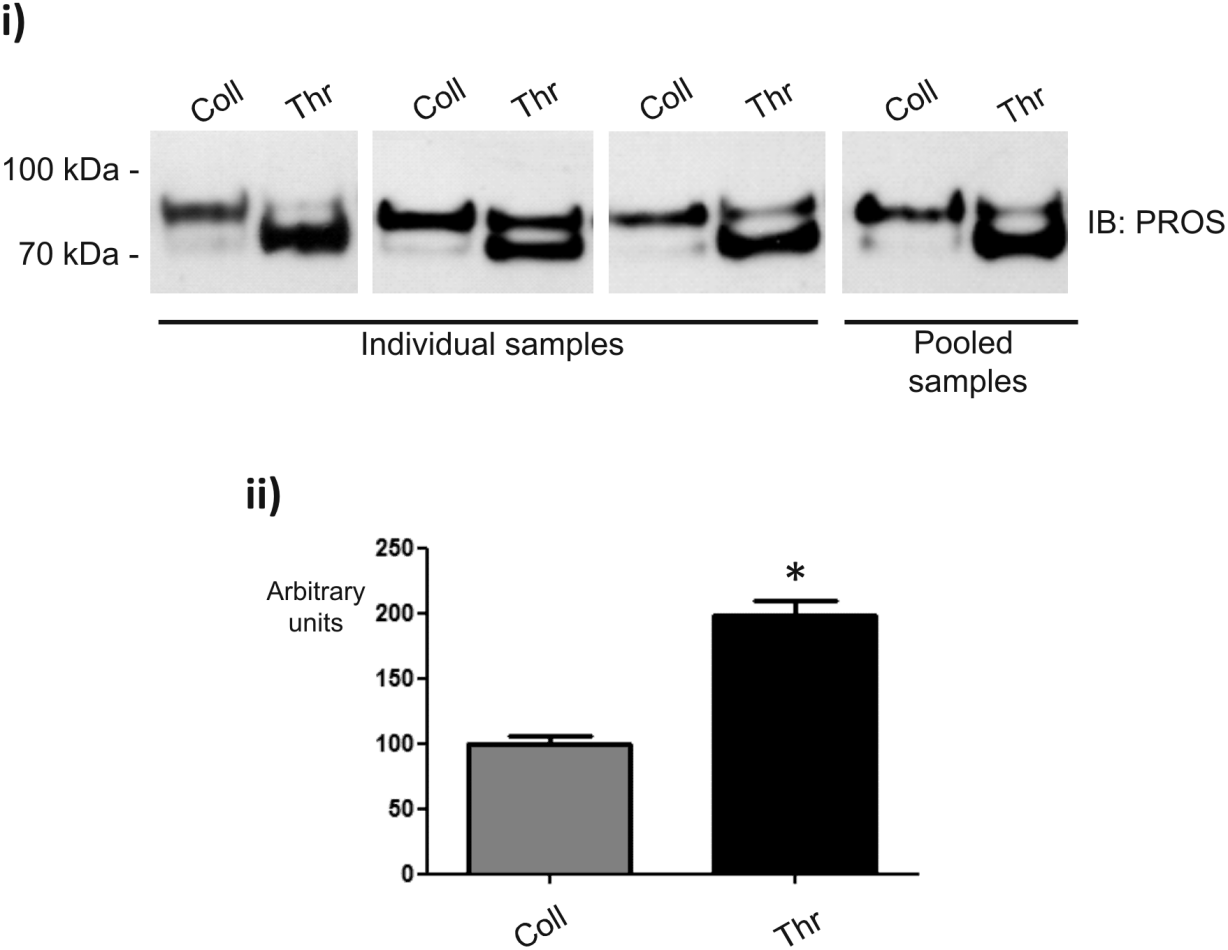
Vitamin K-dependent protein S (PROS) is elevated in the releasate of thrombin-stimulated platelets. Representative western blot images of individual and pooled samples are shown (i) together with densitometry data (ii). Graph show mean values ± SE of band intensities. *p<0.05. IB: immunoblot; Coll: collagen; Thr: thrombin.

Vitamin K-dependent protein S (PROS) is an anticoagulant plasma protein which acts as a cofactor to activated protein C in the degradation of coagulation factors Va and VIIIa. It was found in one spot up-regulated in the thrombin-induced releasate (Fig. 3B). Western blot analyses allowed the detection of PROS in two very close bands at around 75–95 kDa (Fig. 4). The lower band is due to a well described proteolytic action of thrombin on the amino-terminal part of PROS^15^. Overall, PROS was found to be up-regulated in the releasate of thrombin-stimulated platelets, confirming the proteomic results (Fig. 4).

## Discussion

The present study confirms the hypothesis that the platelet releasate may present significant variations depending on the stimulus. To prove so, we chose two potent endogenous agonists that act on different platelet receptors, thrombin and collagen. To make sure differences on the releasate were not due to different agonist power, we chose for both agonists the minimum concentrations that led to equal maximum platelet aggregation after 3 minutes. At those doses, inhibiting PAR-1 or PAR-4 is not sufficient to prevent full thrombin-induced platelet aggregation^9^, whereas we demonstrate GPVI is the main responsible for collagen-induced platelet activation/aggregation. To make sure differences were independent from donors, for each donor both stimulations, with thrombin and collagen, were done and included in the differential analysis. For the 2D-based proteomic analysis we used 24 cm pI 4-7 IPG strips for the first dimension, and large 11%SDS-PAGE gels for the second to increase resolution. Equal amounts of proteins were loaded per gel and DIGE (including an internal standard) was used to increase sensitivity and reproducibility. We acknowledge 2D-DIGE has some limitations (e.g. under-representation of very hydrophobic proteins) and that alternative direct LC-MS/MS proteomic methods, such as label free approaches, would provide additional complementary information. Nevertheless, 2D-DIGE allows a rapid detection of variations in post-translational modifications (PTMs). Indeed, the fact some of the proteins identified are present in the gels in more than one spot highlights the relevance of PTMs (phosphorylations, glycosylations, proteolysis, etc.) in the releasate. More precisely, the amount of identified proteins at a molecular weight lower than the theoretical one in the thrombin-induced releasate highlights the relevance of proteolysis in the secretome induced by this potent endogenous agonist. This issue is of interest and must be studied further. Moreover, the large amount of cytosolic proteins present in the releasate is primarily due to the presence of microvesicles, which were not excluded from the analysis.

The number of differences obtained is quite relevant, which suggests that the platelet receptor initially activated has a clear impact on the secretome, and that the strength of the agonist appears to be important for achieving maximal secretion (in line with data from Coppinger et al.^4^). Thrombin and collagen are powerful agonists and probably produce a strong first wave of platelet secretion, even more relevant than the second wave induced by secondary mediators.

Thus, as mentioned above, the present study reveals a relevant number of differences in the platelet releasate when comparing thrombin (acting primarily on GPCRs such as PAR-1 and PAR-4) and collagen (acting on integrin α2β1 and GPVI) stimulations. These results are also in line with recent studies that show that pro- and anti-angiogenic proteins are organized into separate α-granules and differentially released^16^; moreover, Battinelli and colleagues showed that the release of angiogenesis regulatory proteins from platelet α-granules is modulated by physiological and pathological processes^17^.

One of the most interesting proteins identified, and validated, was Vitamin K-dependent protein S (PROS). This is a plasma protein present in platelet α-granules, which helps to prevent coagulation and stimulate fibrinolysis. As indicated above, western blot analyses allowed the detection of PROS in two very close bands at around 75–95 kDa. Overall, PROS was found to be up-regulated in the releasate of thrombin-stimulated platelets. It is well documented that PROS has an amino-terminal part very sensitive to proteolysis by thrombin^15^, and that is the reason why, in spite of the overall result, the predominant form of PROS detected by western blot in thrombin samples corresponds to the lower band whereas the upper band is predominant in collagen samples. The physiological relevance of PROS function is indicated by a predisposition to venous thrombosis in patients suffering from hereditary PROS deficiency^18^.

### Study limitations

The study has several limitations that should be taken into account. Firstly, due to sample limitations, we had to do the analysis with pooled samples, and took into account protein quantitation prior to pooling. Although recent reports highlighted the proteome of platelets from healthy individuals has a low biological variation and is largely unaffected by aging^19^,^20^, differential releasates between different donors is an issue. Nevertheless, we consider the impact of this issue is smaller among healthy individuals than in pathological circumstances. In addition, we only considered differentially regulated protein spots with a fold change between groups of at least 2. We believe in this way the results should be consistent.

The study has also limitations inherent to 2-DE. Although the chosen proteomic approach allowed the detection of proteome differences due to PTMs (e.g. due to the impact of proteolysis in the thrombin-induced releasate) - which is of interest - it makes more difficult to evaluate differences in total protein abundance. This is because a given protein encoded by an open-reading frame may be present in several spots due to PTMs, some of which may be up- and some down-regulated, even below the established fold change cut-off. As indicated above, a complementary study based on a direct LC-MS/MS proteomic approach would provide additional information of relevance.

Finally, another limitation of the study is the lack of an appropriate loading control for western blotting. This is a typical issue in secretome studies, including platelets'^5^, especially if we have the added factor of the presence of microvesicles in the sample. To address this issue, besides a double protein quantitation, we checked constitutive releasate protein bands that could work as loading controls in 1D stained gels (see Supplementary Figure 1).

In conclusion, we present a high-resolution differential proteome analysis of the platelet releasate and confirm the latter varies depending on the platelet stimulus. Indeed, we demonstrate the existence of significant variations in the platelet releasate between thrombin and collagen stimulations. In addition, we show the primary role of thrombin and collagen receptors in platelet secretion by these agonists, and that GPVI is the primary responsible for collagen-induced platelet activation/aggregation. Overall, our results could have pathological implications given that platelets might be playing a differential role in various diseases and biological processes through the secretion of different subsets of granule proteins and microvesicles following a predominant activation of certain receptors.

## Methods

### Agonists and inhibitors

The following agonists were used: Collagen-related peptide (CRP), with the sequence Gly-Cys-Hyp-(Gly-Pro-Hyp)_10_-Gly-Cys-Hyp-Gly-NH2, was provided by Dr. Richard W. Farndale, from the University of Cambridge (UK), and crosslinked with SPDP (3-(2-pyridyldithio)propionic acid N-hydroxysuccinimide ester) by Dr. Yotis A. Senis, at the University of Birmingham (UK). Thrombin Receptor Activator Peptide 6 (TRAP-6), with the sequence H-Ser-Phe-Leu-Leu-Arg-Asn-OH, was purchased from Bachem. Collagen Reagent HORM® Suspension (KRH) was purchased from Takeda Austria GmbH (Austria). Thrombin was purchased from Sigma (Sigma-Aldrich, St. Louis, MO).

The following inhibitors were used: Fab fragment of the anti-GPVI monoclonal antibody, OM2, was supplied by Otsuka Pharmaceutical Co., Ltd. (Japan). The strong, selective inhibitor of PAR-1, SCH 79797, was purchased from Santa Cruz Biotechnology (Delaware, CA, USA). The selective inhibitor of integrin α2β1, BTT 3033, was from Tocris Bioscience and was provided by Biogen Científica, S.L. (Spain).

### Platelet preparation and activation/inhibition assays

Fresh blood samples were collected from healthy volunteers who had not taken aspirin or antiplatelet medication at least in the previous 10 days. Informed consent was obtained from all volunteers. The study was approved by the local Ethics Committee (Galician Clinical Investigation Ethics Committee) and was in accordance with the Declaration of Helsinki. Blood was collected into 3.2% sodium citrate plastic Vacuette tubes and processed in less than an hour after extraction. Platelets were isolated by an established method in order to limit contamination from other blood cells and plasma proteins^8^. Finally washed platelets were resuspended in Tyrodes-HEPES (134 mmol/L NaCl, 0.34 mmol/L Na_2_HPO_4_, 2.9 mmol/L KCl, 12 mmol/L NaHCO_3_, 20 mmol/L HEPES, 5 mmol/L glucose, 1 mmol/L MgCl_2_, pH 7.3) at 4 × 10^8^ platelets/mL and allowed to rest for 30 minutes at room temperature.

For activations, 500-µL aliquots of platelets were warmed at 37°C for 4 min without stirring and 1 minute with constant stirring at 1200 rpm in a Chrono-log aggregometer, before stimulation for 3 minutes with collagen (30 µg/mL) or thrombin (0.75 U/mL).

When inhibitors were used in aggregation assays, platelets were pre-incubated with the inhibitor for 5 min at 37°C before activation as indicated above. Aggregation curves were recorded.

### Releasate isolation, concentration and precipitation

The platelet releasate was obtained from each donor individually. In order to separate the proteome-releasate from activated platelets two centrifugations were done. Before that, and following platelet activation, prostacyclin was added to each aliquot to a final concentration of 1 μM to prevent spontaneous aggregation during centrifugation. In addition, 10 µL of protease inhibitor cocktail (Sigma-Aldrich, St. Louis, MO) and 2.5 µL of phosphatase inhibitor cocktail (equal parts of benzamidine 1 M, Na_3_VO_4_ 1 M and NaF 100 mM) were added to each aliquot to avoid sample degradation. An initial centrifugation step was carried out at 1000 g for 10 min, followed by a second centrifugation at 10000 g for 2 min, both at 4°C. Supernatants were concentrated in Amicon® Ultra-4, PLBC Ultracel-3 Membrane, 3 kDa (Merck Millipore Ltd. Tullagreen, Carrigtwohill Co. CORK IRL) centrifuging at 4000 g for 1 h and 15 min at 4°C, to a final volume of about 100 µL. Proteins were precipitated in 20% trichloroacetic acid in acetone, as previously described^8^ and finally resuspended in 15 µL of DIGE buffer (65 mM CHAPS, 5 M urea, 2 M thiourea, 0.15 M NDSB-256, 30 mM Tris, 1 mM sodium vanadate, 0.1 mM sodium fluoride, and 1 mM benzamidine). The releasates from each donor were stored at −80°C until they were analyzed.

### 2D-DIGE

Protein quantitation from individual and pooled releasates was done in duplicate with the Coomassie plus protein reagent (Thermo Scientific, Asheville, NC). Seven healthy volunteers participated in the study. Releasates from equal numbers of thrombin and collagen platelet activations were obtained from each donor contributing to the study, as indicated above. A first protein quantitation was done for each sample (collagen or thrombin releasates) from each donor, and equal amounts of protein from donor were pooled for each condition (collagen or thrombin releasates). The pooled samples were again quantitated before DIGE analysis. In total, four gels (technical replicates) were run in the experiment with a total of 150 µg of mixed labelled protein each. These mixed labelled protein contained 50 µg of protein from each sample (collagen and thrombin releasates) randomly labelled with 400 pmol minimal CyDye DIGE fluors (Cy3 and Cy5), and 50 µg of a pool (25 µg from collagen-activated samples and 25 µg from thrombin-activated samples) labelled with 400 pmol Cy2. Labelling was for 30 min on ice in the dark. The reaction was stopped with 1 µl of 10 mM lysine during 10 min on ice in the dark. After that, the three labelled samples were pooled and an equal volume of 2X Sample buffer was added (65 mM CHAPS, 2 M thiourea, 5 M urea, 0.15 M NDSB-256, 130 mM DTT, 4 mM tributylphosphine, 1 mM sodium vanadate, 0.1 mM sodium fluoride, and 1 mM benzamidine). After vortexing, the tube was left 15 min on ice in the dark.

For reswelling, samples were diluted in 2D Sample buffer (5 M urea, 2 M thiourea, 2 mM tributylphosphine, 65 mM DTT, 65 mM CHAPS, 0.15 M NDSB-256, 1 mM sodium vanadate, 0.1 mM sodium fluoride, and 1 mM benzamidine to a final volume of 500 µm), and ampholytes (Servalyt 4–7) were added to a final concentration of 1.6% (v/v). First dimension was on immobilized pH gradient (IPG) strips 4–7, 24 cm (GE Healthcare). Isoelectric focusing (IEF) was in a Multiphor (GE Healthcare) for 64.4 kVhT at 17°C. Following focusing, the IPG strips were immediately equilibrated for 15 min in reduction buffer (6 M urea, 50 mM tris pH 8.8, 30% glycerol, 2% w/v SDS, 65 mM DTT and traces of bromophenol blue) in the dark and then for 15 min in alkylation buffer (6 M urea, 50 mM tris pH 8.8, 30% glycerol, 2% w/v SDS, 135 mM iodoacetamide and traces of bromophenol blue) with gentle agitation. IPG strips were washed out with ultrapure water and placed on top of the second dimension gels, embedded with 0.5% melted agarose. Proteins were separated in the second dimension by SDS-polyacrylamide gel electrophoresis (PAGE) on 11% gels at run conditions of 10°C, 20 mA per gel for 1 h, followed by 40 mA per gel for 4 h by using an Ettan Dalt 6 system (GE Healthcare). Following electrophoresis gels were scanned directly in a Typhoon 9410 (GE Healthcare).

### Differential image analysis

Gels images were processed with Progenesis SameSpots (v 4.5) from Nonlinear Dynamics Ltd. (Newcastle, UK) in order to find real differences between both conditions of study. Both manual and automatic alignments were used to align the images. SameSpots detects the spots simultaneously across all images generating a master gel list containing all the features. This master gel list is then applied to all the aligned gels which prevents the loss of any data points and should in principle increase the precision of spot volume estimates. Internal calibration of the 2D-DIGE gel images with regard to pI and molecular weight was carried out with SameSpots built-in tools. All gels were compared with each other and fold values as well as p-values of all spots were calculated by SamesSpots software using one way ANOVA analysis. Differential expression of a protein present in the gels was considered significant when the fold change was at least 2 and the p value was below 0.05.

### MS analysis

Differentially regulated spots were excised manually from the gels and in-gel digested with trypsin following the protocol defined by Shevchenko with minor modifications^21^. Identifications were by MALDI-TOF or MALDI-TOF/TOF, in a 4800 Plus MALDI-TOF/TOF analyzer (Applied Biosystems), and by LC–MS/MS on an EASY-nLC (Proxeon, Bruker Daltonik) and a Bruker Amazon ETD ion trap.

MALDI-MS and MS/MS data were combined through the GPS Explorer Software Version 3.6 to search a non-redundant protein database (Swissprot 2012_10) using the Mascot software version 2.2 (Matrix Science). For LC-MS/MS, database search was performed with the Mascot v2.4 search tool (Matrix Science, London, UK) screening SwissProt (release_56.1). Further details on the MS protocol can be found in the Supplementary Methods.

### Western blotting

For western blotting validations, proteins were separated by 10% SDS-PAGE, loading 4 µg of protein per lane in each gel. Following electrophoresis, proteins were transferred onto polyvinylidene difluoride (PVDF) membranes (GE Healthcare). The membranes were blocked in 5% BSA in TBS-T (20 mM Tris-HCl (pH 7.6), 150 mM NaCl and 0.1% Tween 20) overnight at 4°C and incubated for 90 min at room temperature with the following Santa Cruz Biotechnology, Inc. (Delaware, CA, USA) antibody: mouse anti-Protein S (sc-271326), dilution 1:500. Following washes in TBS-T, membranes were exposed to horseradish peroxidase-labeled goat anti-mouse, or donkey anti-goat antibodies (dilution 1:5000) (Pierce, Rockford, IL). Membranes were washed again and processed using an enhanced-chemiluminiscence system (ECL, Pierce, Rockford, USA). Bands intensity was quantified by densitometry. Densitometry was performed using ImageJ (National Institute of Health, Bethesda, MD, USA) version 1.47, and statistical analysis was by Mann-Whitney test comparing groups peer to peer.

## Author Contributions

P.V. performed experiments, analyzed data and contributed to writing the paper; I.I. and I.R. performed experiments; A.G. designed research, analyzed data, provided vital reagents and analytical tools, and wrote the paper. All authors reviewed the manuscript.

## Supplementary Material

Supplementary Information Supplementary Information

## Figures and Tables

**Table 1:** 2D-DIGE analysis: List of proteins identified in spots that vary when comparing the releasate of thrombin- and collagen-activated platelets. Fibrinogen spots are excluded. All variant protein spots have a fold change cutoff ≥2 and p<0.05. Red font numbers correspond to spots with increased intensity in the thrombin-induced releasate whereas green font numbers correspond to spots increased in the collagen-induced releasate. Further information can be found in Supplementary Table 1

Protein	Uniprot Code	Spot No.
Actin, cytoplasmic 2	ACTG_HUMAN	815
Alpha-2-HS-glycoprotein	FETUA_HUMAN	687
Amyloid beta A4 protein	A4_HUMAN	1843
Apolipoprotein A-I	APOA1_HUMAN	1397, 1413
Apolipoprotein D	APOD_HUMAN	1393
Caspase-3	CASP3_HUMAN	1199
Cardiotrophin-like cytokine factor 1	CLCF1_HUM	1309
Clusterin	CLUS_HUMAN	961, 973, 1111, 1393
Coagulation factor V	FA5_HUMAN	1559
Glutathione peroxidase 1	GPX1_HUMAN	1438, 1516
Glutathione S-transferase P	GSTP1_HUMAN	1397
Glutathione synthetase	GSHB_HUMAN	1860
Haloacid dehalogenase-like hydrolase domain-containing protein 2	HDHD2_HUMAN	1274
Inter-alpha-trypsin inhibitor heavy chain H2	ITIH2_HUMAN	672, 1818, 1842, 1843, 1869
Ig lambda-2 chain C regions	LAC2_HUMAN	1301
Ig kappa chain C region	IGKC_HUMAN	1301
Metalloproteinase inhibitor 1	TIMP1_HUMAN	1274
Multimerin-1	MMRN1_HUMAN	1244, 1309
Myosin regulatory light polypeptide 9	MYL9_HUMAN	1560
Nidogen-2	NID2_HUMAN	1203
Nucleosome assembly protein 1-like 1	NP1L1_HUMAN	680
POTE ankyrin domain family member E	POTEE_HUMAN	815
Proteasome subunit beta type-6	PSB6_HUMAN	1378
Putative beta-actin-like protein 3	ACTBM_HUMAN	815
Ras-related protein Rap-1A	RAP1A_HUMAN	1585
Rho GDP-dissociation inhibitor 1	GDIR1_HUMAN	1348, 1847
Rho GDP-dissociation inhibitor 2	GDIR2_HUMAN	1373
Serpin B6	SPB6_HUMAN	1672
Serum albumin	ALBU_HUMAN	800, 819, 1275
SH3 domain-binding glutamic acid-rich-like protein 3	SH3L3_HUMAN	1730
Thioredoxin-like protein 1	TXNL1_HUMAN	1111
Thrombospondin-1	TSP1_HUMAN	1226
Transgelin-2	TAGL2_HUMAN	1508
Transthyretin	TTHY_HUMAN	1866
Tubulin beta-1 chain	TBB1_HUMAN	775, 776, 778
Vitamin K-dependent protein S (PROS)	PROS_HUMAN	1839
von Willebrand factor	VWF_HUMAN	1818
