# Supplementary material for: A 2D-DIGE-based proteomic analysis reveals differences in the platelet releasate composition when comparing thrombin and collagen stimulations
Source: Sci Rep. 2015 Feb 3;5:8198. doi: 10.1038/srep08198 (PMC4316189; doi:10.1038/srep08198)
Supplement: Supplementary Information [file srep08198-s1.pdf]

A 2D-DIGE-based proteomic analysis reveals differences in the platelet releasate composition when comparing thrombin and collagen stimulations

Paula Vélez[1,2,3], Irene Izquierdo[1,2,3], Isaac Rosa[1,2,3] and Ángel García*[1,2,3]

[1]Center for Research in Molecular Medicine and Chronic Diseases (CIMUS), Universidade de Santiago de Compostela, Santiago de Compostela, Spain;

[2]Instituto de Investigación Sanitaria (IDIS), Santiago de Compostela, Spain;

[3]Departament of Pharmacology, Faculty of Pharmacy, Universidade de Santiago de Compostela, Santiago de Compostela, Spain.

Supplementary Figure 1

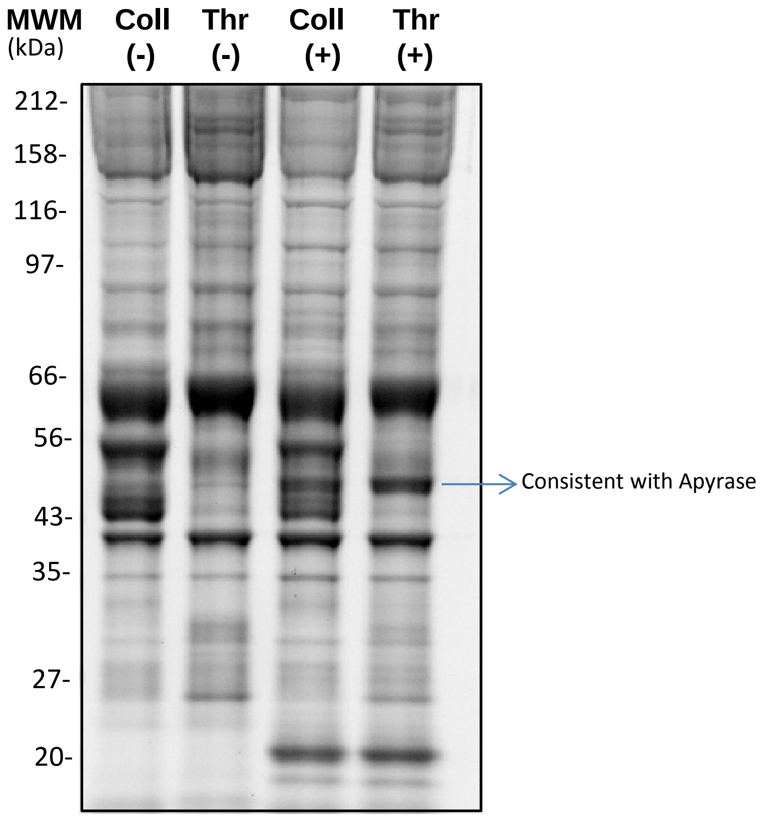

Supplementary Figure 1. Releasate profile comparison following platelet activation with thrombin and collagen in presence and absence of inhibitors of secondary mediators and integrilin (αIIbβ3 inhibitor). Platelets were activated for 3 min with 0.75 U/mL thrombin or 30 µg/mL collagen. When indicated, platelets were pre-incubated for 5 min with the following inhibitors: 2 U/mL apyrase, 10 µM indomethacin, and 9 µM integrilin. Releasates were obtained as indicated in the Methods section. Secreted proteins were separated by 1D-SDS-PAGE in 4-12% Nu-PAGE Bis-Tris gels (Invitrogen). Ten micrograms of protein were loaded per lane. The gel was stained with Sypro Ruby fluorescent dye (Lonza). MWM: molecular weight marker; Coll(-): collagen without inhibitors; Thr(-): thrombin without inhibitors; Coll(+): collagen with inhibitors; Thr(+): thrombin with inhibitors

Supplementary Table 1. Additional MS data.

Spot	N† / %‡	Mass score	Peptides identified by MS		Identified protein	UniProt Code	MW (estimated)/ MW (theo)	pI (estimated/ pI (theo)	Fold change
			m/z	Sequence					
477	--	--	--	--	Not identified	--	--	--	4.6
481	--	--	--	--	Not identified	--	--	--	2.3
672	6/5	167.0	729.459<br>845.478<br>1337.725<br>1419.724<br>1465.816<br>1582.859	IQKNVK<br>TILDDLR<br>FYNQVSTPLLR<br>VQFELHYQEVK<br>KFYNQVSTPLLR<br>IQPSGGTNINEALLR	Inter-alpha-trypsin inhibitor heavy chain H2[aa]	ITIH2_HUMAN	78.953/ 106.463	5.89/6.40	3.6
680	4/14	79.0	1336.634<br>1356.723<br>1859.976<br>2244.144	FYEEVHDLER<br>YAVLYQPLFDK<br>LDGLVETPTGYIESLPR<br>QLTVQMMQNPQILAALQER	Nucleosome assembly protein 1-like 1[aa]	NP1L1_HUMAN	77.161/45.374	4.51/4.63	2.3
687	4/13.1	170.3	554.3190<br>407.1541<br>424.2111<br>699.6625	EHAVEGDCDFQLLK<br>FSVVYAK<br>CNLLAEK<br>HTFMGVVSLGSPSGEVSHPR	Alpha-2-HS-glycoprotein	FETUA_HUMAN	73.578/39.324	4.59/5.43	2.1
688	--	--	--	--	Not identified	--	--	--	2.5
716	--	--	--	--	Not identified	--	--	--	-2.3
775	10/29	169.0	1077.529<br>1130.598<br>1142.611<br>1159.627<br>1334.645<br>1415.749<br>1631.829<br>1695.842<br>2284.175<br>2312.136	IREEYPDR<br>FPGQLNADLR<br>YLTVACIFR<br>LAVNMVPFPR<br>ISVYYNEAYGR<br>EVDQQLLSVQTR<br>AVLVDLEPGTMDSIR<br>ALSVAELTQQMFDAR<br>GLSMAATFIGNNTAIQEIFNR<br>LHFFMPGFAPLTAQGSQQYR	Tubulin beta-1 chain[aa]	TBB1_HUMAN	52.373/50.326	5.24/5.05	-4.6
	11/32	101.0	759.448<br>1034.533<br>1117.536<br>1150.518<br>1293.753<br>1491.749<br>1513.730<br>1682.960<br>2011.958<br>2207.092<br>2536.255	IIPFNR<br>VGPEADKYR<br>VELEDWNGR<br>TSTADYAMFK<br>QSGLYFIKPLK<br>YEASILTHDSSIR<br>YLQEIYNSNNQK<br>IHLISTQSAIPYALR<br>CHAGHLNGVYYQGGTYSK<br>EGFGHLSPTGTTEFWLGNEK<br>AIQLTYNPDESSKPNMIDAATLK	Fibrinogen gamma chain[aa]	FIBG_HUMAN	52.373/51.511	5.24/5.37	
776	14/39	364.0	944.518	GASALQLER	Tubulin beta-1 chain[aa]	TBB1_HUMAN	52.373/50.326	5.28/5.05	-2.5

			1077.534<br>1130.600<br>1142.605<br>1159.627<br>1287.716<br>1334.645<br>1415.753<br>1631.838<br>1695.840<br>2028.032<br>2284.168<br>2312.123<br>2336.995	IREEYPDR<br>FPGQLNADLR<br>YLTVACIFR<br>LAVNMVPFPR<br>KLAVNMVPFPR<br>ISVYYNEAYGR<br>EVDQQLLSVQTR<br>AVLVDLEPGTMDSIR<br>ALSVAELTQQMFDAR<br>GHYTEGAELIENVLEVVR<br>GLSMAATFIGNNTAIQEIFNR<br>LHFFMPGFAPLTAQGSQQYR<br>AVLEEDEEVTEEAEMEPEDK					
778	17/54	504.0	808.356<br>944.518<br>1077.535<br>1130.601<br>1142.606<br>1159.628<br>1198.562<br>1334.642<br>1415.754<br>1631.836<br>1695.839<br>2028.041<br>2284.165<br>2296.151<br>2336.991<br>2707.389<br>3808.836	EEYPDR<br>GASALQLER<br>IREEYPDR<br>FPGQLNADLR<br>YLTVACIFR<br>LAVNMVPFPR<br>VSEHFSAMFK<br>ISVYYNEAYGR<br>EVDQQLLSVQTR<br>AVLVDLEPGTMDSIR<br>ALSVAELTQQMFDAR<br>GHYTEGAELIENVLEVVR<br>GLSMAATFIGNNTAIQEIFNR<br>LHFFMPGFAPLTAQGSQQYR<br>AVLEEDEEVTEEAEMEPEDK<br>LTTPTYGDLNHLVSLTMSGITTSLR<br>HESESCDCLQGFQIVHSLGGGTGSGMGTLLMN<br>KIR	Tubulin beta-1 chain[a]	TBB1_HUMAN	52.075/50.326	5.20/5.05	-2.4
800	5/10	53.0	960.563<br>1358.639<br>1467.835<br>1910.929<br>2045.092	FQNALLVR<br>AVMDDFAAFVEK<br>RHPDYSVVLLLR<br>RPCFSALEVDETYVPK<br>VFDEFKPLVEEPQNLIK	Serum albumin[aa]	ALBU_HUMAN	47.395/69.366	6.01/5.92	-5.4
815	9/33	313.0	795.468<br>976.452<br>1132.531<br>1516.714<br>1639.797<br>1790.898<br>1954.056<br>1962.914<br>2359.144	IIAPPER<br>AGFAGDDAPR<br>GYSFTTTAER<br>QEYDESGPSIVHR<br>LDLAGRDLTDYLMK<br>SYELPDGQVITIGNER<br>VAPEEHPVLLTEAPLNPK<br>YPIEHGIVTNWDDMEK<br>KDLYANTVLSGGTTMYPGIADR	Actin cytoplasmic 2[aa]	ACTG_HUMAN	46.514/41.792	5.25/5.31	-2.7

	5/7.8	175.0	976.452<br>1516.714<br>1790.898<br>2280.143<br>2720.284	AGFAGDDAPR<br>QEYDESGPSIVHR<br>SYELPDGQVITIGNER<br>MVVEVDSMPAASSVKKPFGLR<br>DLYTNTVLSGGTTMYPGMAHRMQK	POTE ankyrin domain family member E[aa]	POTEE_HUMAN	46.514/121.363	5.25/5.83	
	3/13.3	174.0	1189.629<br>1516.714<br>1790.898	EITALAPSIMK<br>QEYDESGPSIVHR<br>SYELPDGQVITIGNER	Putative beta-actin-like protein 3[aa]	ACTBM_HUMAN	46.514/42.016	5.25/5.91	
819	7/15	103.0	960.561<br>1149.574<br>1358.640<br>1467.836<br>1910.933<br>2045.095<br>2260.034	FQNALLVR<br>DAHKSEVAHR<br>AVMDDFAAFVEK<br>RHPDYSVVLLLR<br>RPCFSALEVDETYVPK<br>VFDEFKPLVEEPQNLIK<br>EFNAETFTFHADICTLSEK	Serum albumin[aa]	ALBU_HUMAN	46.394/69.366	5.77/5.92	-11.5
855	--	--	--	--	Not identified	--	--	--	-3.9
899	--	--	--	--	Not identified	--	--	--	-2.5
956	--	--	--	--	Not identified	--	--	--	-3.7
961	3/7	164.0	779.403<br>1288.643<br>1873.992	ALQEYR<br>ELDESLQVAER<br>LFDSDPITVTVPVEVSR	Clusterin[aa]	CLUS_HUMAN	40.827/52.494	4.70/5.88	-3.2
962	--	--	--	--	Not identified	--	--	--	2.6
973	3/6	140.0	732.402<br>1075.577<br>1393.699	SGSGLVGR<br>RPHFFFPK<br>ASSIIDELFQDR	Clusterin[aa]	CLUS_HUMAN	40.547/52.494	4.75/5.88	-4.2
1086	--	--	--	--	Not identified	--	--	--	3.4
1103	--	--	--	--	Not identified	--	--	--	-9.0
1111	8/33.9	480.1	733.6163<br>584.7448<br>631.2872<br>818.3149<br>637.1970<br>680.8106<br>572.7898<br>565.2346	VGVKPVGSDPDFQPELSGAGSR<br>IAPAFSSMSNK<br>VRIDQYQGADAVGLEEK<br>IDQYQGADAVGLEEK<br>QHLENDPGSNEDTDIPK<br>GYMDLMPFINK<br>FQGPDNGQGPK<br>SMDFEEAER	Thioredoxin-like protein 1	TXNL1_HUMAN	35.301/32.251	4.99/4.84	-4.7
	2/7.3	92.2	536.3023<br>771.9748	EIQNAVNGVK<br>VTTVASHTSDSDVPSGVTEVVVK	Clusterin	CLUS_HUMAN	35.301/52.494	4.99/5.88	
1199	1/4.0	65.0	559.6930	SGTDVDAANLR	Caspase-3	CASP3_HUMAN	31.937/31.607	6.43/6.09	-2.2
1203	7/7	291.0	1299.698<br>1330.694<br>1376.711<br>1417.748<br>1759.839<br>2083.019<br>2129.066	EDTSPAVLGLAAR<br>LANPLHFYEAR<br>IETSSLDGENRR<br>ESYNVQLQLPAR<br>SEGPYFSLTSTEQSVK<br>SHLYGITAVYPYCPTGRK<br>FSNLYVGTNGIISTQDFPR	Nidogen-2[aa]	NID2_HUMAN	31.657/151.253	4.88/5.09	2.5

1226	8/8	355.0	989.616<br>1030.505<br>1062.566<br>1207.637<br>1394.738<br>1616.819<br>1864.908<br>2195.072	GFLLLASLR<br>GPDPSSPAFR<br>FQDLVDAVR<br>SITLFVQEDR<br>FVFGTTPEDILR<br>GGVNDNFQGVLQNVR<br>MENAELDVPIQSVFTR<br>IPESGGDNSVFDIFELTGAAR	Thrombospondin-1[aa]	TSP1_HUMAN	30.736/129.382	5.60/4.71	-2.0
1244	5/14.8	284.9	846.0040<br>497.8626<br>757.2810<br>647.3497<br>440.6205	AIQLTYNPDESSKPNMIDAATLK<br>YEASILTHDSSIR<br>YLQEIYNSNNQK<br>QSGLYFIKPLK<br>LDGSVDFK	Fibrinogen gamma chain	FIBG_HUMAN	29.694/51.511	5.57/5.37	-4.3
	1/1.1	74.0	716.8663	LVEENALAPDFSK	Multimerin-1	MMRN1_HUMAN	29.694/138.110	5.57/8.15	
1263	--	--	--	--	Not identified	--	--	--	2.7
1269	--	--	--	--	Not identified	--	--	--	2.1
1271	--	--	--	--	Not identified	--	--	--	5.2
1274	2/10.1	123.9	617.2464<br>497.2080	GFQALGDAADIR<br>SEEFLIAGK	Metalloproteinase inhibitor 1	TIMP1_HUMAN	28.373/23.170	6.01/8.46	2.4
	1/6.6	75.2	851.8697	DDVGGAQDVGMLGILVK	Haloacid dehalogenase-like hydrolase domain-containing protein 2	HDHD2_HUMAN	28.373/28.535	6.01/5.84	
1275	7/13	57.0	927.495<br>960.563<br>1149.610<br>1467.839<br>1742.849<br>1910.921<br>2045.053	YLYEIAR<br>FQNALLVR<br>LVNEVTEFAK<br>RHPDYSVVLLLR<br>HPYFYAPELLFFAK<br>RPCFSALEVDETYVPK<br>VFDEFKPLVEEPQNLIK	Serum albumin[aa]	ALBU_HUMAN	28.333/69.366	5.71/5.92	3.2
1278	--	--	--	--	Not identified	--	--	--	2.4
1292	--	--	--	--	Not identified	--	--	--	3.0
1301	2/32.1	111.2	712.5689<br>752.3000	VDNALQSGNSQESVTEQDSK<br>DSTYSLSSTLTLSK	Ig kappa chain C region	IGKC_HUMAN	27.532/11.608	5.42/5.58	2.1
	1/17.9	57.3	993.4045	AAPSVTLFPPSSEELQANK	Ig lambda-2 chain C regions	LAC2_HUMAN	27.532/11.293	5.42/6.91	
1309	2/7.5	76.5	845.9940<br>647.3155	AIQLTYNPDESSKPNMIDAATLK<br>QSGLYFIKPLK	Fibrinogen gamma chain	FIBG_HUMAN	27.051/51.511	5.98/5.37	-3.5
	1/1.1	81.3	716.7845	LVEENALAPDFSK	Multimerin-1	MMRN1_HUMAN	27.051/138.110	5.98/8.15	
	1/3.6	40.7	428.6747	LGAETLPR	Cardiotrophin-like cytokine factor 1	CLCF1_HUMAN	27.051/24.086	5.98/8.91	
1348	6/27.9	398.8	601.7407<br>959.3426<br>485.5411<br>809.2749<br>631.1764<br>876.3069	SIQEIQELDK<br>SIQEIQELDKDDESLR<br>QSFVLKEGVEYR<br>IDKTDYMVGSYGPR<br>TDYMVGSYGPR<br>AEEYEFLTPVEEAPK	Rho GDP-dissociation inhibitor 1	GDIR1_HUMAN	25.009/23.207	5.03/5.01	8.9

1373	5/25.9	397.8	970.2622<br>854.8027<br>656.2696<br>716.6494<br>428.2652	APEPHVEEDDDDELDSK<br>ELQEMDKDDESLIK<br>TLLGDGPVVTDPK<br>TLLGDGPVVTDPKAPNVVVTR<br>APNVVVTR	Rho GDP-dissociation inhibitor 2	GDIR2_HUMAN	24.591/22.988	5.23/5.08	3.1
1378	2/13.0	199.6	542.3423<br>558.2401<br>555.7220	TTTGSYIANR<br>LAAIAESGVER<br>QVLLGDQIPK	Proteasome subunit beta type-6	PSB6_HUMAN	24.519/25.357	4.94/4.80	8.3
1393	3/14.3	145.1	517.7174<br>436.2565<br>615.7708	IPTTFENGR<br>VLNQELR<br>NILTSNNIDVK	Apolipoprotein D	APOD_HUMAN	24.290/21.275	5.05/5.06	3.6
	1/5.1	60.2	771.9686	VTTVASHTSDSDVPSGVTEVVVK	Clusterin	CLUS_HUMAN	24.290/52.494	5.05/5.88	
1397	4/15.7	296.7	908.2943<br>700.7830<br>806.8016<br>608.2316	DSGRDYVSQFEGSALGK<br>DYVSQFEGSALGK<br>LLDNWDSVTSTFSK<br>ATEHLSTLSEK	Apolipoprotein A-I	APOA1_HUMAN	24.218/30.777	5.42/5.56	-3.9
	1/5.2	47.3	669.3233	PPYTVVYFPVR	Glutathione S-transferase P	GSTP1_HUMAN	24.218/23.355	5.42/5.43	
1413	9/31.8	555.2	908.3457<br>700.7926<br>806.8114<br>626.7458<br>734.3038<br>437.1680<br>524.1869<br>651.2347<br>608.2018	DSGRDYVSQFEGSALGK<br>DYVSQFEGSALGK<br>LLDNWDSVTSTFSK<br>VQPYLDDFQK<br>VEPLRAELQEGAR<br>AELQEGAR<br>LSPLGEEMR<br>THLAPYSDELR<br>ATEHLSTLSEK	Apolipoprotein A-I	APOA1_HUMAN	23.976/30.777	5.30/5.56	3.8
1416	--	--	--	--	Not identified	--	--	--	5.9
1438	2/10	58.0	1169.673<br>1313.588	FLVGPDGVPLR<br>DYTQMNELQR	Glutathione peroxidase 1[aa]	GPX1_HUMAN	23.627/22.088	5.72/6.15	3.4
1461	--	--	--	--	Not identified	--	--	--	3.2
1508	7/49	75.0	820.430<br>1218.627<br>1231.686<br>1279.603<br>1415.605<br>1694.818<br>3346.679	GPAYGLSR<br>NVIGLQMGTNR<br>TLMNLGGLAVAR<br>NFSDNQLQEGK<br>GASQAGMTGYGMPR<br>QMEQISQFLQAAER<br>ENFQNWLKDGTVLCELINALYPEGQAPVK	Transgelin-2[aa]	TAGL2_HUMAN	22.288/22.391	5.63/8.41	2.6
1516	6/32.5	330.4	657.1674<br>530.2144<br>669.0071<br>929.8311<br>585.3046<br>442.5360	DYTQMNELQR<br>NEEILNSLK<br>YVRPGGGFEPNFMLFEK<br>EALPAPSDDATALMTDPK<br>FLVGPDGVPLR<br>FLVGPDGVPLRR	Glutathione peroxidase 1	GPX1_HUMAN	22.192/22.088	5.73/6.15	2.9
1530	--	--	--	--	Not identified	--	--	--	3.3
1540	--	--	--	--	Not identified	--	--	--	-3.9

1553	--	--	--	--	Not identified	--	--	--	6.0
1558	--	--	--	--	Not identified	--	--	--	10.2
1559	2/1	114.0	1894.914<br>2293.081	AADIEQQAVFAVFDENK<br>LELQGCEVNGCSTPLGMENGK	Coagulation factor V[aa]	FA5_HUMAN	21.348/251.703	4.69/5.68	10.9
1560	4/28	238.0	1246.587<br>1253.558<br>1449.585<br>2106.977	GNFNYVEFTR<br>EAFNMIDQNR<br>FTDEEVDEMYR<br>ATSNVFAMFDQSQIQEFK	Myosin regulatory light polypeptide 9[aa]	MYL9_HUMAN	21.336/19.827	4.72/4.78	8.5
1585	1/6.0	59.8	493.2444	LVVLGSGGVGK.	Ras-related protein Rap-1A	RAP1A_HUMAN	20.853/20.987	4.94/6.93	3.0
1595	--	--	--	--	Not identified	--	--	--	4.8
1647	--	--	--	--	Not identified	--	--	--	4.2
1650	--	--	--	--	Not identified	--	--	--	9.1
1654	--	--	--	--	Not identified	--	--	--	6.2
1657	--	--	--	--	Not identified	--	--	--	7.4
1658	--	--	--	--	Not identified	--	--	--	12.9
1663	--	--	--	--	Not identified	--	--	--	3.4
1669	--	--	--	--	Not identified	--	--	--	4.7
1672	1/4.0	85.2	801.7602	ADFSGMSQTDLSLSK	Serpin B6[aa]	SPB6_HUMAN	19.093/42.621	4.48/5.0	4.3
1673	--	--	--	--	Not identified	--	--	--	16.6
1674	--	--	--	--	Not identified	--	--	--	18.1
1684	--	--	--	--	Not identified	--	--	--	17.7
1727	--	--	--	--	Not identified	--	--	--	-4.1
1730	1/10.8	56.4	528.7684	VYSTSVTGSR	SH3 domain-binding glutamic acid-rich-like protein 3[aa]	SH3L3_HUMAN	17.374/10.437	4.55/4.7	4.3
1738	--	--	--	--	Not identified	--	--	--	5.6
1739	--	--	--	--	Not identified	--	--	--	4.1
1753	--	--	--	--	Not identified	--	--	--	2.2
1800	--	--	--	--	Not identified	--	--	--	-2.8
1818	14/6	221.0	705.392<br>767.403<br>845.482<br>977.511<br>1015.597<br>1097.493<br>1169.600<br>1312.656<br>1406.679<br>1520.772<br>1700.733<br>1738.872<br>2408.111<br>2864.336	LECRK<br>STSVFAR<br>TNTGLALR<br>YPPGTSLSR<br>AVSPLPYLR<br>AFVVDMMER<br>LSGEAYGFVAR<br>SFSIIGDFQNGK<br>GLYLETEAGYYK<br>IDGSGNFQVLLSDR<br>LSYGEDLQMDWDGR<br>YFTFSGICQYLLAR<br>CLPSACEVVTGSPRGDSQSSWK<br>VPLDSSPATCHNNIMKQTMVDSSCR	von Willebrand factor[aa]	VWF_HUMAN	148.539/309.264	4.52/5.29	2.1
	4/4	103.0	845.482<br>1337.724	TILDDLR<br>FYNQVSTPLLR	Inter-alpha-trypsin inhibitor heavy chain H2[aa]	ITIH2_HUMAN	148.539/106.463	4.52/6.40	

			1419.719<br>1582.854	VQFELHYQEVK<br>IQPSGGTNINEALLR					
1825	--	--	--	--	Not identified	--	--	--	-8.6
1838	--	--	--	--	Not identified	--	--	--	5.4
1839	3/4	63.0	956.500<br>1018.466<br>1749.945	VYFAGFPR<br>FSAEFDFR<br>SQDILLSVENTVIYR	Vitamin K-dependent protein S[aa]	PROS_HUMAN	128.828/75.122	4.80/5.48	2.0
1842	5/5	141.0	792.433<br>845.478<br>1419.723<br>1465.799<br>1582.859	ALYAQAR<br>TILDDLR<br>VQFELHYQEVK<br>KFYNQVSTPLLR<br>IQPSGGTNINEALLR	Inter-alpha-trypsin inhibitor heavy chain H2[aa]	ITIH2_HUMAN	155.109/106.463	4.61/6.40	4.5
1843	15/21	163.0	824.423<br>829.431<br>904.463<br>963.460<br>987.501<br>1099.587<br>1144.543<br>1228.617<br>1266.672<br>1374.651<br>1462.596<br>1755.846<br>1914.860<br>1996.901<br>2022.151	HFEHVR<br>FLHQER<br>HVFNMLK<br>VEAMLNDR<br>SQVMTHLR<br>AVIQHFQEK<br>WYFDVTEGK<br>QQLVETHMAR<br>THPHFVIPYR<br>VESLEQEAANER<br>CAPFFYGGCGGNR<br>ISYGNDALMPSLTETK<br>YLETPGDENEHAHFQK<br>EQNYSDDVLANMISEPR<br>LALENYITALQAVPPRPR	Amyloid beta A4 protein[aa]	A4_HUMAN	149.136/83.943	4.49/4.73	2.4
	5/5	144.0	732.398<br>845.481<br>1337.721<br>1419.725<br>1582.852	TQVADAK<br>TILDDLR<br>FYNQVSTPLLR<br>VQFELHYQEVK<br>IQPSGGTNINEALLR	Inter-alpha-trypsin inhibitor heavy chain H2[aa]	ITIH2_HUMAN	149.136/106.463	4.49/6.40	
1860	5/15.0	296.1	845.9940<br>497.8477<br>757.2816<br>647.3559<br>559.2233	AIQLTYNPDESSKPNMIDAATLK<br>YEASILTHDSSIR<br>YLQEIYNSNNQK<br>QSGLYFIKPLK<br>VELEDWNGR	Fibrinogen gamma chain	FIBG_HUMAN	47.275/51.511	5.78/5.37	-3.7
	4/10.8	222.3	882.4243<br>514.7682<br>912.8290<br>485.6713	QIEINTISASFGGLASR<br>AIENELLAR<br>EGGGNNLYGEEMVQALK<br>ASYILMEK	Glutathione synthetase	GSHB_HUMAN	47.275/52.384	5.78/5.67	
1866	2/24.5	124.2	683.8579<br>817.9774	GSPAINVAVHVFR<br>ALGISPFHEHAEVVFTANDSGPR	Transthyretin[aa]	TTHY_HUMAN	18.960/15.887	4.94/5.49	2.6
1867	--	--	--	--	Not identified	--	--	--	2.9
1869	6/5	152.0	732.395	TQVADAK	Inter-alpha-trypsin inhibitor heavy chain H2[aa]	ITIH2_HUMAN	77.460/106.463	5.99/6.40	4.7

			845.480<br>1337.718<br>1419.719<br>1465.803<br>1582.852	TILDDLR<br>FYNQVSTPLLR<br>VQFELHYQEVK<br>KFYNQVSTPLLR<br>IQPSGGTNINEALLR					
1874	3/20.6	176.7	639.9150<br>631.1940<br>876.3501	SIQEIQELDKDDESLR<br>TDYMVGSYGPR<br>AEEYEFLTPVEEAPK	Rho GDP-dissociation inhibitor 1	GDIR1_HUMAN	25.370/23.207	4.92/5.01	6.7
1875	--	--	--	--	Not identified	--	--	--	-6.3
1876	--	--	--	--	Not identified	--	--	--	-3.1

Identifications were by ESI-TRAP unless stated otherwise. Proteins identified by [a]MALDI-MS or [aa]MALDI-MS/MS. † Number of matched peptides. ‡ % coverage of full length protein by tryptic peptides. All differential proteins have a p value lower than 0.05. A positive fold change indicates that the protein feature is up-regulated in the releasate of thrombin-stimulated platelets, whereas a negative fold change indicates that the protein feature is up-regulated in the platelet-collagen releasate.

Supplementary Methods

MS analysis

For MALDI analysis, dried peptides were dissolved in 4 µL of 0.5% formic acid. Equal volumes (0.5 µL) of peptide and matrix solution (3 mg alpha-cyano-4-hydroxycinnamic acid (α-CHCA) dissolved in 1 mL of 50% acetonitrile in 0.1% trifluoroacetic acid) were deposited using the thin layer method, onto a 384 Opti-TOF 123x81 mm MALDI plate (Applied Biosystems) and allowed to dry at room temperature. Mass spectrometric data were obtained in an automated analysis loop using a 4800 MALDI-TOF/ TOF analyzer (Applied Biosystems). Spectra were acquired in the reflector positive-ion mode with a Nd:YAG, 355nm wavelength laser, at 200 Hz laser frequency, and 1000 to 2000 individual spectra were averaged. The experiments were acquired uniform with fixed laser intensity. For MS/MS 1kV analysis mode, precursors were accelerated to 8 kV in source 1, selected with a relative resolution of 200 (FWHM) and metastable suppression. Fragment ions generated by collision with air in a CID chamber were further accelerated by 15 kV in source 2. Automated analysis of mass data was performed using the 4000 Series Explorer Software version 3.5.3 (Applied Biosystems). Internal calibration of MALDI-TOF mass spectra was performed using two trypsin autolysis ions with m/z = 842.510 and m/z = 2211.105. For MALDI-MS/MS, calibrations were performed with fragment ion spectra obtained for Glub-fibrinopeptide (4700 Cal Mix, Applied Biosystems). MALDI-MS and MS/MS data were combined through the GPS Explorer Software Version 3.6 to search a non-redundant protein database (Swissprot 2012_10) using the Mascot software version 2.2 (Matrix Science), with 50 ppm precursor tolerance, 0.6 Da MS/MS fragment tolerance, CAM (carbamidomethyl cystein) as fixed modification, oxidized methionine as variable modification and allowing 1 missed cleavage. MALDI-MS(/MS) spectra and database search results were manually inspected in detail using the previous software. For combined MS and MS/MS data, identifications were accepted

when Confidence Interval (C.I.%) of GPS software was 95% or higher. Since Protein Scores and Ion Scores from different searches cannot be directly compared, GPS software calculates this C.I.% in order to combine results from MS and MS/MS database searches. This coefficient value means that the probability that the observed match is a random event is lower than 5%. For PMF spectra, identifications were also accepted when (C.I.%) of GPS software was 99% or higher.

For LC-MS/MS digested peptide mixtures dissolved in 0.1% formic acid were separated in an EASY-nLC (Proxeon, Bruker Daltonik GmbH) with a reverse phase nanocolumn (Easy column SC200 C18 3µm 120A 360 µm OD/75µm ID, L=10cm) from Proxeon. Ionized peptides were analyzed in a Bruker Amazon ETD ion trap. Automated analysis of mass data was achieved by Data Analysis 4.0 and BioTools 3.2 from Bruker Daltonik GmbH. Database search was performed with the Mascot v2.4 search tool (Matrix Science, London, UK) screening SwissProt (release_56.1). Searches were restricted to human taxonomy allowing carbamidomethyl cysteine as a fixed modification and oxidized methionine as potential variable modification. Both the precursor mass tolerance and the MS/MS tolerance were set at 0.3 and 0.4 Da, respectively, allowing 1 missed tryptic cleavage site. All spectra and database results were manually inspected in detail using the above software, especially in the case of identifications based on one peptide hit. For the latter, positive identification by MS was only accepted when more than 50% y-ions were obtained for a peptide comprising at least eight amino acids long and no missed tryptic cleavage site. Positive hits corresponded to Mascot scores > 40 plus the fulfillment of the above criteria.
